# Measuring wealth in rural communities: Lessons from the Sanitation, Hygiene, Infant Nutrition Efficacy (SHINE) trial

**DOI:** 10.1371/journal.pone.0199393

**Published:** 2018-06-28

**Authors:** Bernard Chasekwa, John A. Maluccio, Robert Ntozini, Lawrence H. Moulton, Fan Wu, Laura E. Smith, Cynthia R. Matare, Rebecca J. Stoltzfus, Mduduzi N. N. Mbuya, James M. Tielsch, Stephanie L. Martin, Andrew D. Jones, Jean H. Humphrey, Katherine Fielding, the SHINE Trial Team

**Affiliations:** 1 Zvitambo Institute for Maternal and Child Health Research, Harare, Zimbabwe; 2 Department of Economics, Middlebury College, Middlebury, VT, United States of America; 3 Department of International Health, Johns Hopkins Bloomberg School of Public Health, Baltimore, MD, United States of America; 4 Program in International Nutrition, Division of Nutritional Sciences, Cornell University, Ithaca, NY, United States of America; 5 Department of Epidemiology and Environmental Health, School of Public Health and Health Professions, University at Buffalo, State University of New York, Buffalo, NY, United States of America; 6 Global Alliance for Improved Nutrition (GAIN), Washington, DC, United States of America; 7 Department of Global Health, Milken Institute School of Public Health, George Washington University, Washington, DC, United States of America; 8 Department of Nutrition, Gillings School of Global Public Health, University of North Carolina Chapel Hill, Chapel Hill, NC, United States of America; 9 Department of Nutritional Sciences, School of Public Health, University of Michigan, Ann Arbor, MI, United States of America; 10 Department of Infectious Disease Epidemiology, London School of Hygiene and Tropical Medicine, London, United Kingdom; Institut de recherche pour le developpement, FRANCE

## Abstract

**Background:**

Poverty and human capital development are inextricably linked and therefore research on human capital typically incorporates measures of economic well-being. In the context of randomized trials of health interventions, for example, such measures are used to: 1) assess baseline balance; 2) estimate covariate-adjusted analyses; and 3) conduct subgroup analyses. Many factors characterize economic well-being, however, and analysts often generate summary measures such as indices of household socio-economic status or wealth. In this paper, a household wealth index is developed and tested for participants in the cluster-randomized Sanitation, Hygiene, Infant Nutrition Efficacy (SHINE) trial in rural Zimbabwe.

**Methods:**

Building on the approach used in the Zimbabwe Demographic and Health Survey (ZDHS), we combined a set of housing characteristics, ownership of assets and agricultural resources into a wealth index using principal component analysis (PCA) on binary variables. The index was assessed for internal and external validity. Its sensitivity was examined considering an expanded set of variables and an alternative statistical approach of polychoric PCA. Correlation between indices was determined using the Spearman’s rank correlation coefficient and agreement between quintiles using a linear weighted Kappa statistic. Using the 2015 ZDHS data, we constructed a separate index and applied the loadings resulting from that analysis to the SHINE study population, to compare the wealth distribution in the SHINE study with rural Zimbabwe.

**Results:**

The derived indices using the different methods were highly correlated (r>0.9), and the wealth quintiles derived from the different indices had substantial to near perfect agreement (linear weighted Kappa>0.7). The indices were strongly associated with a range of assets and other wealth measures, indicating both internal and external validity. Households in SHINE were modestly wealthier than the overall population of households in rural Zimbabwe.

**Conclusion:**

The SHINE wealth index developed here is a valid and robust measure of wealth in the sample.

## Introduction

Poverty and human capital development—including nutrition, health and education—are inextricably linked [1]. Therefore, research on human capital typically collects measures of economic well-being and incorporates them into analyses. For example, studies of health outcomes commonly include an index of socio-economic status (SES) as a key covariate [2]. Such indices can reflect economic well-being better than a single asset or component, and use fewer degrees of freedom in statistical models compared with multiple assets [3].

A number of approaches have been developed to measure SES in health studies [4]. Direct measures of income or consumption expenditure are widely used in developed countries [5] and, when available, are usually preferred to constructed indices using more distal variables [6]. Measurement of income, however, can be difficult in low-income or developing countries, particularly in rural settings where it can vary considerably throughout the year and where much of the population participates in agriculture and the informal economy [6]. Consumption expenditure is an attractive alternative and typically more stable throughout the year [7], but also difficult to measure for developing country households because of the prevalence of own production and in-kind transactions, lack of detailed expenditure accounts and potential irregular large expenditures such as healthcare [8]. Accordingly, reliable income or consumption expenditure data require relatively complex and costly survey instruments.

An alternative approach to directly measuring income or expenditures is the construction of an asset-based wealth index; typically, such indices are derived from a long list of common household possessions and access to and quality of water, sanitation and housing. This approach is used in most Demographic and Health Surveys (DHS) [6] to estimate relative wealth within the study population. Asset ownership is easier to measure reliably than income or consumption expenditures [9], and is generally regarded as a good indicator of long-term household wealth [3, 6, 10]. There are a variety of approaches for aggregating household assets and characteristics into a single metric.

The importance of measuring economic well-being is not limited to observational analyses using multi-purpose surveys like the DHS, but also includes other study designs such as randomized trials of interventions and programs. In that context, wealth indices offer a powerful way to incorporate economic well-being when: 1) assessing baseline balance; 2) estimating covariate-adjusted analyses to reduce bias and increase precision; and 3) conducting subgroup analyses or examining potential moderating effects.

Using baseline data from the Sanitation, Hygiene, Infant Nutrition Efficacy (SHINE) Trial conducted in rural Zimbabwe between 2012 and 2017 [11], we developed and validated a household wealth index. For validation, first we grouped the index into quintiles and examine means of variables included and not included in the index across the quintiles. Second, we compared the extent to which the index categorized relative wealth of members of the study population similarly to categorizations based on index measures constructed using alternative approaches. Third, we constructed a separate wealth index using data from the 2015 Zimbabwe Demographic and Health Survey (ZDHS) and applied it to the SHINE study population, to compare the wealth distributions in the SHINE study population with the rest of rural Zimbabwe. The index will be used to adjust for relative wealth in analyses of the SHINE trial [11].

## Background

We conducted a review of methods used to estimate a household-level asset-based wealth index in low-income countries from 1995–2015 (Table 1). The review focussed on which housing characteristics and possessions different studies included and the methodologies used for combining them into an index.

**Table 1:** Summary of published examples of household-level asset-based wealth indices for low-income settings.

Citation	Country	Study setting	Brief description of variables included	Method^1^	Purpose for derived index
Amek N, Vounatsou P, Obonyo B, Hamel M, Odhiambo F, Slutsker L, et al. Using health and demographic surveillance system (HDSS) data to analyze geographical distribution of socio-economic status; an experience from KEMRI/CDC HDSS. Acta Trop. 2015;144:24–30.	Kenya	Rural	Occupation of household head, primary source of drinking water, use of cooking fuel, ownership of lantern lamp, sofa, radio, bicycles and television and ownership of livestock including poultry, pigs, donkey, cattle, sheep and goats.	PCA, Polychoric PCA, MCA	Outcome as socio-economic status discriminatory tool
Balen J, McManus DP, Li YS, Zhao ZY, Yuan LP, Utzinger J, et al. Comparison of two approaches for measuring household wealth via an asset-based index in rural and peri-urban settings of Hunan province, China. Emerg Themes Epidemiol. 2010;7(1):7.	China	Rural & Peri-urban	Ownership of land, animals, rice cooker, microwave, VCR, satellite dish, phone, motorbike, refrigerator, washing machine and boat, along with indicators of floor type, roof type, toilet type, whether medicines at home and measure of overcrowding.	PCA, FA	Outcome in estimating socio-economic position
Boccia D, Hargreaves J, Ayles H, Fielding K, Simwinga M, Godfrey-Faussett P. Tuberculosis infection in Zambia: the association with relative wealth. Am J Trop Med Hyg. 2009;80(6):1004–11.	Zambia	Rural & Urban	Indicators of floor type, roof type, type of water supply, electricity and distance to market. Weekly number of meals containing proteins and number of coping strategies.	PCA	Explanatory variable in associations with tuberculosis
Booysen F, Van der Berg S, Burger R, Von Maltitz M, du Rand G. Using an asset index to assess trends in poverty in seven Sub-Saharan African countries. World Dev. 2008;36(6):1113–30.	Ghana, Kenya, Mali, Senegal, Tanzania, Zambia, Zimbabwe	Rural & Urban	Ownership of radio, television, refrigerator and bicycle; sanitation type, floor type and the main water source type.	MCA	Outcome in poverty assessment
Hargreaves JR, Morison LA, Gear JS, Kim JC, Makhubele MB, Porter JD, et al. Assessing household wealth in health studies in developing countries: a comparison of participatory wealth ranking and survey techniques from rural South Africa. Emerg Themes Epidemiol. 2007;4:4.	South Africa	Rural	Value of selected non-livestock assets, value of livestock assets, land tenure, wall type, type of toilet, electricity, quality of water supply, density (persons/room), proportion receiving regular income, education and gender of household head, regularity of having a meal of mealie meal alone, bread alone or worse	PCA	Outcome in assessing household wealth
Kennedy G, Nantel G, Brouwer ID, Kok FJ. Does living in an urban environment confer advantages for childhood nutritional status? Analysis of disparities in nutritional status by wealth and residence in Angola, Central African Republic and Senegal. Public Health Nutr. 2006;9(2):187–93.	Angola, Central African Republic, Senegal	Rural & Urban	Household access to electricity, radio or television; household ownership of bicycle, motorcycle or car; type of material of dwelling floor; number of rooms in the dwelling; main source of drinking water; and type of toilet facility	PCA	Explanatory variable in associations with undernutrition
Kimuna SR, Djamba YK. Wealth and Extramarital Sex Among Men in Zambia. International Family Planning Perspectives. 2005;31(2):83–9.	Zambia	Rural & Urban	Ownership of radio, television, refrigerator, bicycle, car or truck and electricity	Simple additive score	Explanatory variable in associations with extra-marital sex
Kongnyuy EJ, Wiysonge CS, Mbu RE, Nana P, Kouam L. Wealth and sexual behaviour among men in Cameroon. BMC Int Health Hum Rights. 2006;6:11.	Cameroon	Rural & Urban	A score was assigned to each household amenity (details of actual amenities included not provided)	Simple additive score	Explanatory variable in associations with sexual behavior
Luby SP, Halder AK. Associations among handwashing indicators, wealth, and symptoms of childhood respiratory illness in urban Bangladesh. Trop Med Int Health. 2008;13(6):835–44.	Bangladesh	Urban	Floor type, wall type, roof type, number of living rooms; ownership of fan, radio, television, cycle, refrigerator, mobile phone; cooking fuel type, mother’s education	PCA	Confounding variable in relationship between handwashing and childhood respiratory illness
Uthman OA, Kongnyuy EJ. A multilevel analysis of effect of neighbourhood and individual wealth status on sexual behaviour among women: evidence from Nigeria 2003 Demographic and Health Survey. BMC Int Health Hum Rights. 2008;8:9.	Nigeria	Rural & Urban	A score was assigned to each household amenity (details of actual amenities included not provided)	Simple additive score	Explanatory variable in associations with extra-marital sex
Schellenberg JA, Victora CG, Mushi A, de Savigny D, Schellenberg D, Mshinda H, et al. Inequities among the very poor: health care for children in rural southern Tanzania. Lancet. 2003;361(9357):561–6.	Tanzania	Rural	Ownership of chickens or ducks, other animals, radio, bicycle, tin roof, mosquito nets, house occupied, whether household head has other sources of income apart from farming, education of household head	PCA	Outcome in classifying by socio-economic status

^1^PCA–Principal Component Analysis; MCA–Multiple Correspondence Analysis; FA–Factor Analysis

Researchers have used a wide range of variables to construct wealth indices, including ownership of durable or other assets, housing characteristics, sanitary facilities and access to such services as electricity and drinking water. The set of variables included differs across studies, in large part reflecting data availability but also the relevance of different variables in different settings [12–14]. For the DHS, Rutstein and Johnson [6], and Rutstein [15] recommend the inclusion of any asset that can reflect economic status.

Alongside, researchers have developed a number of methods for combining the components. While some use simple additive scales, most employ methods that give more valuable or important assets relatively more weight. One approach uses the inverse of the proportion of the survey population possessing the particular asset, essentially assuming that less common assets are more valuable and therefore more likely to be owned by wealthier households [16]. A disadvantage of this method is that some assets may not exhibit a clear linear (or even monotonic) relationship between frequency of ownership and wealth over the entire wealth distribution of a given population [17]. Another approach is to weight each household asset according to its current monetary value [16]; this method can be difficult to implement in rural settings where the value of assets such as housing or land may be difficult to determine.

One of the most common methods used for assigning weights to household assets in wealth index construction is principal component analysis (PCA), a statistical method used to reduce a set of variables into a smaller set that are linear combinations of the original variables capturing maximal variation [18, 19]. By construction, the resulting components are uncorrelated with one another and therefore regarded as reflecting different dimensions of wealth [18]. The first combination (the first principal component) is usually used in the construction of the index because it contains the most information common to all the variables [3]. Several of the studies in Table 1 use PCA as their main approach [20–24]. Moreover, the DHS [6, 15], World Bank country reports on health, nutrition, population and poverty [7], and the Multiple Indicator Cluster Surveys (MICS) of the United Nations Children’s Fund (UNICEF) [25] all use this method.

PCA is not ideal when data are discrete or categorical, however, because this violates the normality assumption underlying the method. Kolenikov and Angeles [26] recommend performing PCA on the polychoric correlations of binary variables. The polychoric correlation assumes that each of the variables is influenced by a latent, normally distributed variable and estimates the correlation between them (via maximum likelihood). PCA is then performed on the polychoric correlation matrix of variables that are no longer binary [27]. A third method, Multiple Correspondence Analysis (MCA) is designed for categorical variables. MCA estimates associations between categories of two or more categorical variables using contingency tables [28].

A final method less commonly employed in this literature is Factor Analysis (FA). FA utilizes only the variance that is common among the original variables as opposed to PCA which utilizes all of the variance [29]. FA is used when the analyst assumes a causal model exists in which latent constructs determine a set of observable variables. The goal is to explain the common variance among the observable variables that arises from their relationship to the latent constructs. Balen et al. [30] find that PCA and FA yield similar results when they compared the two approaches for constructing a wealth index.

## Methods

### The SHINE trial

The SHINE trial was conducted in two contiguous rural districts of Midlands Province in central Zimbabwe where 65% of working adults were employed in the agricultural sector primarily as small-scale farmers [31]. In brief, SHINE was a cluster-randomized community-based 2x2 factorial trial testing the independent and combined effects of protecting babies from fecal ingestion through a water, sanitation and hygiene [WASH] intervention and optimizing nutritional adequacy of infant diet through an infant and young child feeding [IYCF] intervention. Primary outcomes, measured at 18 months of age, were length-for-age Z-score (LAZ) and hemoglobin concentration[11]. Clusters were defined as the catchment area of between 1–4 village health workers (VHW) from the Zimbabwean Ministry of Health and Child Care (MoHCC). A total of 212 clusters were allocated to one of the four treatment groups (Standard of Care [SOC] alone, SOC+WASH, SOC+IYCF or SOC+WASH+IYCF) at a public randomization using a highly constrained randomization technique. Between November 2012 and March 2015, 5,280 pregnant women were identified through prospective pregnancy surveillance and enrolled at a median of 12 (interquartile range [IQR] 9–16) weeks gestation.

Research nurses collected baseline data during home visits, about 2 weeks after enrollment. By design, the SHINE baseline survey drew heavily from the standard ZDHS instrument and, therefore, most of the variables used in the construction of the ZDHS wealth index were available in the baseline, as well as some additional ones specifically added to capture local conditions.

### Development and assessment of SHINE wealth index

We constructed the SHINE wealth index based on the index developed for the 2010–11 ZDHS [32] and following the general approach utilized for DHS [6, 15], with modifications made to suit the SHINE study data, region and objectives. Our primary analysis was based on PCA using a core set of household assets and characteristics all coded as binary indicator variables. Factor loadings from the first principal component for each item were standardized so that each has mean of zero and standard deviation (SD) of one. A wealth index for each household was calculated by adding the standardized loadings for all assets in the set (Eq 1).

$${WI}_{i}=\sum_{k}\alpha_{k}\beta_{ik}$$ (1)

Where *α_k_* is the loading for asset *k*, and $\beta_{ik}=(x_{ik}-{\bar{x}}_{k})/s_{k}$ with *x_ik_* = 1 if household *i* owns asset *k*, or 0 if household *i* does not own asset *k*. ${\bar{x}}_{k}$ and *s_k_* are the sample mean and SD for asset k for all households.

We refer to the resulting index as the SHINE wealth index. In addition, we conducted two sensitivity analyses: 1) PCA using an expanded set of household characteristics (expanded SHINE wealth index); and 2) polychoric PCA. Lastly, using the 2015 ZDHS data we conducted PCA restricted to rural households to enable a comparison of the distribution of the two samples using a single common set of weights, and provide further validation of the approach.

### Statistical methods

Variable selection for the primary analysis for the SHINE wealth index was based on all variables used in the 2010–11 ZDHS wealth index that were also available in the SHINE study. All variables were recoded as binary and those with frequencies < 4% or > 96% were excluded. This cut-off was used to exclude particularly uncommon assets while ensuring inclusion of vehicles, an important asset in this rural context. We also excluded variables closely linked with the principal hypotheses of the SHINE intervention, such as, latrine availability, so that future analysis of the trial can better isolate their association with outcomes or explore them as effect moderators (Table 2). Those variables remaining were defined as the core set.

**Table 2:** 2010–11 ZDHS wealth index components compared to SHINE wealth index^1^.

Item	ZDHS	Available in SHINE baseline	Included in SHINE index	Rationale and modifications
**Housing Characteristics**				
Main floor material (8 binary categories)	✓	✓	✓	Included, but combined into single binary indicator for higher quality floor material
Main exterior wall material (12 binary categories)	✓	✓	✓	Included, but combined into single binary indicator for higher quality wall material
Main roof material (10 binary categories)	✓	✓	✓	Included, but combined into single binary indicator for higher quality roof material
Electricity	✓	✓	✓	Included
Cooking fuel type (8 binary categories)	✓	✓	✗	Not included, < 2% use any fuel other than wood in baseline
Type of water source (13 binary categories)	✓	✓	✗	Not included, to permit examination of moderating effects
Toilet/Latrine (10 binary categories)	✓	✓	✗	Not included, to permit examination of moderating effects
Share toilet with other households	✓	✓	✗	Not included, to permit examination of moderating effects
Share latrine with other households	✓	✓	✗	Not included, to permit examination of moderating effects
HH members per sleeping room	✓	✗	✗	Not included, number of rooms used for sleeping unavailable in baseline
**Ownership of household durable goods (binary)**				
Radio	✓	✓	✓	Included
Television	✓	✓	✓	Included
Refrigerator	✓	✓	✗	Not included, < 3% own in baseline. ZDHS indicates < 3% own in rural Midlands Province
Bicycle	✓	✓	✓	Included
Motorcyle	✓	✓	✗	Not included, < 1% own in baseline. ZDHS indicates < 1% own in rural Midlands Province
Car/Truck	✓	✓	✓	Included
Phone (landline)	✓	✓	✓	Included, but combined landline and cell phone
Phone (cell)	✓	✓	✓	
Watch/Clock	✓	✓	✓	Included
Boat with motor	✓	✗	✗	Not included, unavailable in baseline but also not relevant in region without large bodies of water. ZDHS indicates 0% own in rural Midlands Province
Solar panel	✓	✓	✓	Included
Generator	✓	✓	✗	Not included, < 4% own in baseline. ZDHS indicates ~ 15% own in rural Midlands Province
Computer	✓	✗	✗	Not included, unavailable in baseline. ZDHS indicates < 1% own in rural Midlands Province
Bank account	✓	✗	✗	Not included, unavailable in baseline. ZDHS indicates ~ 20% have in rural Midlands Province
**Agricultural resources and equipment**				
Owns land for agriculture	✓	✓	✗	State owned land under communal control, ~ 90% have access
Acres of land for agriculture	✓	✓	✗	As above
Tractor	✓	✓	✗	Not included, < 3% own in baseline. ZDHS indicates < 1% own in rural Midlands Province
Animal drawn cart	✓	✓	✓	Included
Wheelbarrow	✓	✓	✓	Included
**Ownership of animals**				
Owns livestock, horses or farm animals	✓	✓	✗	Not included, use specific categories only (listed below)
Cattle	✓	✓	✓	Included
Goats	✓	✓	✓	Included, combined goat and sheep
Sheep	✓	✓	✓	
Chicken or other poultry	✓	✓	✓	Included
Horses/Donkeys/Mules	✓	✗	✗	Not included, available in baseline only in non-specific "other" category for these animals less commonly owned in study region.
Horses	✓	✗	✗	As above. ZDHS indicates < 2% own in rural Midlands Province
Donkeys/mules	✓	✗	✗	As above. ZDHS indicates ~ 10% own in rural Midlands Province
Pigs	✓	✗	✗	As above. ZDHS indicates ~ 4% own in rural Midlands Province
Rabbits	✓	✗	✗	As above. ZDHS indicates < 3% own in rural Midlands Province

^1^ ✓indicates available or used.

✗indicates unavailable or not used.

In our primary analysis, we carried out PCA using the set of core binary variables and present the proportion explained by the first principal component and the loadings. Scree plot was used to determine the number of components required. We also computed the Hofmann’s index of complexity for each item and the overall mean to check adequacy of the retained principal components (Eq 2) [33].

$$c_{k}=\frac{{\left(\sum_{j}\alpha_{jk}^{2}\right)}^{2}}{\sum_{j}\alpha_{jk}^{4}}$$ (2)

where *α*_jk_ is the loading on the j-th principal component for the k-th asset.

Only data from households with five or fewer missing values in the core variables were included, and missing data were imputed by multiple imputation using the ‘imputePCA’ function of the R package ‘psych’ [34]. Internal validity was assessed by grouping the index into quintiles and performing the non-parametric test for trend on the means of the variables included in the index across the quintiles. External validity was assessed similarly, using measures associated with wealth but not included in the index [3]. These included measures of income and expenditures over the last month, coping strategies related to food security [35], and indicators of household dietary diversity [36].

In the first sensitivity analysis, we carried out a separate PCA analysis using an expanded set of binary variables including 1) variables used in the 2010–11 ZDHS wealth index, but excluded from the core set of variables due to their being included in the SHINE interventions and 2) variables not used in the 2010–11 ZDHS wealth index but available in the SHINE survey, including other locally relevant assets. The second sensitivity analysis used polychoric PCA with its theoretically better statistical properties for binary data on the “core” set of variables [26]. Missing data were imputed by multiple imputation using the ‘MICE’ function of the R package ‘missMDA’ [37]. We estimated the tetrachoric correlations among the binary variables and then carried out PCA on the correlation matrix.

We estimated Spearman rank correlation coefficients and their 95% confidence intervals calculated via percentiles based on 1,000 bootstrap repetitions for the SHINE wealth index with (i) the expanded SHINE wealth index and (ii) the polychoric PCA index. We also calculated, for these two comparisons and using the sample for the expanded index, the percentage of observations in agreement, and the linear weighted kappa statistics, comparing quintiles, quartiles and terciles for each index to assess sensitivity using standard cut-offs [38]. We calculated 95% confidence intervals of the weighted Kappa statistics via percentiles based on 1,000 bootstrap repetitions [39].

Finally, we estimated a separate PCA on rural households for all of Zimbabwe, using the 2015 ZDHS implemented from July to December 2015 [40]. We based it on the “core” variables common to the 2015 ZDHS and the SHINE wealth index. Using the estimated loadings from the first principal component on the 15 common items (ownership of a wheelbarrow, used in the SHINE index, was unavailable in the 2015 ZDHS), we predicted index scores for the (in-sample) rural ZDHS households. We then used those same loadings for the first principal component from the ZDHS and the distribution of the variables from the SHINE households to estimate a new index for (out-of-sample) SHINE households. This enabled a comparison of the distribution of the two samples using a single common set of weights.

Wilcoxon rank sum tests were used to compare medians of non-normally distributed continuous variables and Chi square tests were used to compare proportions for categorical variables and trend analyses across derived quintiles [41]. Multiple imputations and calculation of the Hofmann’s index were done in R [42] and all remaining analyses conducted in Stata 14 [43].

### Ethics

The Medical Research Council of Zimbabwe (IRB # MRCZ-A-1675) and the Institutional Review Board of the Johns Hopkins Bloomberg School of Public Health (IRB # 00004205) provided initial and ongoing review and approval of the SHINE study protocol (Clinical Trials Registration: NCT01824940). All participants provided written informed consent. The London School of Hygiene and Tropical Medicine Research Ethics Committee gave consent for this analysis (Reference 9338) for the work conducted for a MSc dissertation [44].

## Results

Of the 39 variables found in the 2010–11 ZDHS wealth index describing housing characteristics, ownership of assets and agricultural resources, 30 were available in some form in the SHINE baseline survey (Table 2). Of these, 18 were included in the SHINE wealth index selected as described in the Table; landline and cell phone, and goats and sheep were both regrouped as single variables, resulting in 16 variables in total. Excluded from the index were five variables because they had minimal variation, four variables because they will be used for direct exploration of moderating effects in the SHINE trial, and three variables were less relevant in the SHINE study district. The latter included, for example, land “ownership” in an area where nearly all households have access to (state-owned) land, but under communal control—in this context land is a poor indicator of wealth.

SHINE consented 5,280 women, of whom 4,704 (89.1%) were available for the baseline visit. In brief, those available for the visit were older, median (IQR) 25.3 (20.4–31.1) years compared to those who were not available, median (IQR) 22.9 (19.4–28.8) years, p<0.001; of higher parity, 2 (1–3) compared to 1 (0–2), p <0.001; had higher proportion married %(n), 95.6 (4,267) compared to 88.4 (229), p<0.001). There was no evidence of difference in education years (p = 0.798) and size of household (p = 0.460) between those who were available for the visit and those who were not available. Few households had electricity from the power grid, the majority owned a radio and cellphone (usually powered via solar charger) and about one-third owned a television (usually powered via battery) (Table 3). Nearly two-fifths owned a bicycle, but very few had a vehicle. Reflecting the predominantly agricultural nature of economic opportunity in this rural area, the vast majority of households cultivated crops (primarily maize), more than one half owned cattle and sheep, and nearly 80% raised chickens or other poultry.

**Table 3:** Means of variables in SHINE wealth index (16 variables).

Item	Mean (%)	N^a^
*Housing Characteristics (binary)* Higher quality floor material	50.3	4,590
Higher quality floor material	50.3	4,590
Higher quality wall material	66.0	4,595
Higher quality roof material	12.5	4,599
Electricity	7.4	4,649
*Ownership of household durable goods* Radio	68.5	4,656
Television	32.9	4,657
Radio	68.5	4,656
Bicycle	38.6	4,657
Car/Truck	4.2	4,646
Phone (landline or cell)	89.5	4,662
Watch/Clock	13.5	4,654
Solar panel (typically to charge phone)	65.3	4,655
*Ownership of agricultural resources and equipment*		
Animal drawn cart	32.1	4,658
Wheelbarrow	48.8	4,657
*Ownership of animals*		
Cattle	57.4	4,658
Goats or sheep	52.3	4,658
Chicken or other poultry	79.0	4,657

^a^Notes: 4,704 households had at least some baseline data available; 4,665 had five or fewer missing values for variables in this table.

**Fig 1:**
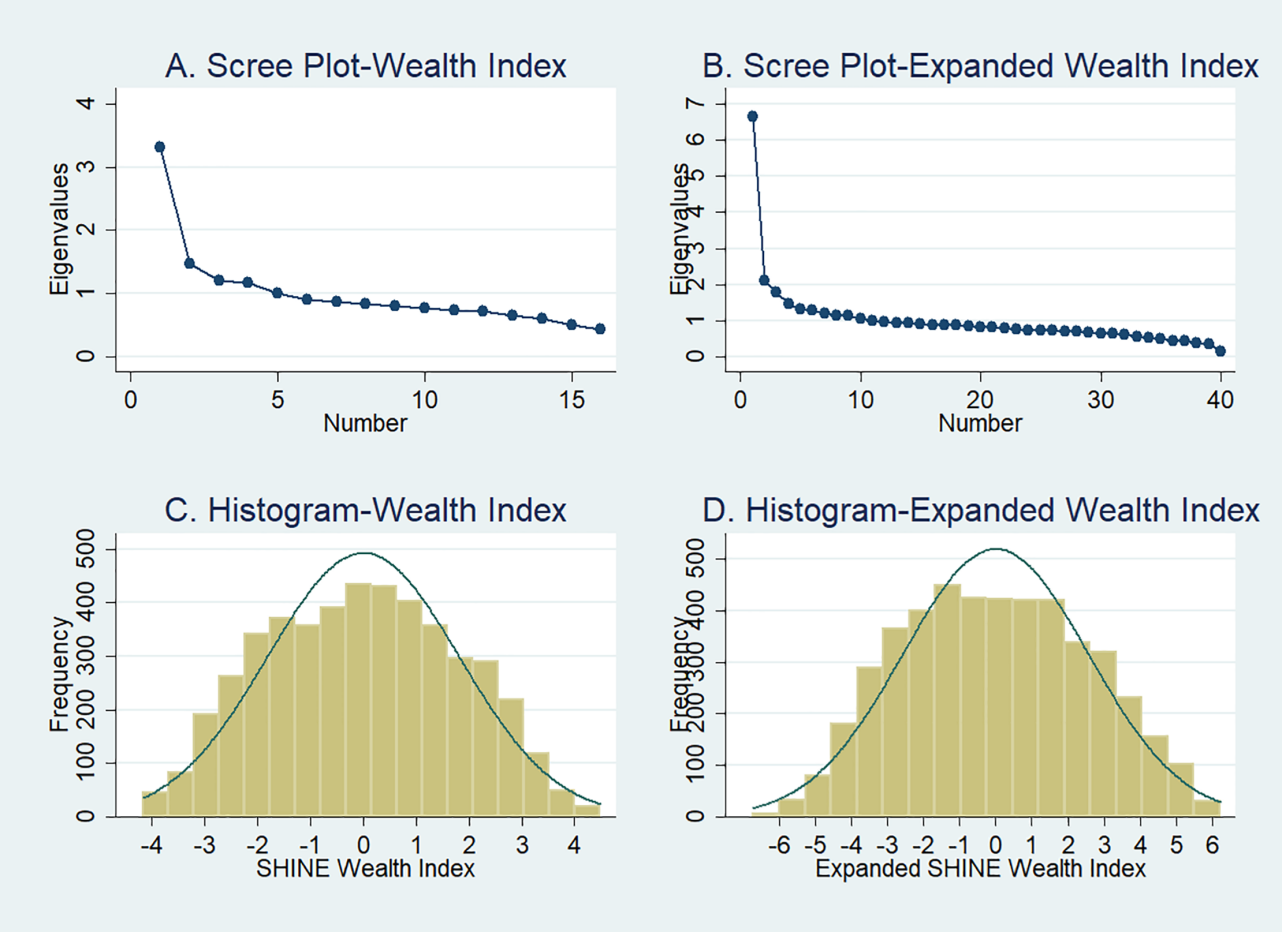
Scree plots of eigenvalues based on core set of 16 variables (A) and expanded set of 40 variables (B); histograms of standardized household wealth indices based on core set of 16 variables and (C) and expanded set of 40 variables (D).

Data from 4,665 women, who had five or fewer missing values for the core variables, were used to construct the SHINE wealth index using PCA on 16 binary variables (Table 4, Fig 1). Overall, 3.5% of this sample had one or more imputations, with most of those having just one missing value imputed. The scree plot shows substantial levelling of eigenvalues after the first principal component, which explained 21% of the variation (Fig 1A). The selected model retained two principal components. The overall mean item complexity was 1.4 supporting adequacy of the model. All loadings were positive and all but four (of the 16) greater than 0.2 (Table 4). The median loading was 0.24 (IQR, 0.20–0.30). The predicted wealth index scores based on the first principal component suggest an approximately symmetric, and normal, distribution for households in the sample (Fig 1C). There was relatively little truncation or clumping: no more than 1% of the observations had any single index score value (the maximum was 43 of 4,665).

**Table 4:** Principal component analysis (PCA) for SHINE wealth indices.

Item	SHINE Wealth Index 16 variables		Expanded SHINE Wealth Index 40 variables	
	First principal component loadings	Hofmann’s index of complexity	First principal component loadings	Hofmann’s index of complexity
*Common assets*				
Higher quality floor material	0.292	1.8	0.188	1.7
Higher quality wall material	0.187	1.2	0.118	1.8
Higher quality roof material	0.151	1.9	0.084	1.6
Electricity	0.092	1.2	0.058	1.8
Radio	0.284	1.0	0.160	1.4
Television	0.299	1.4	0.183	2.0
Bicycle	0.236	1.6	0.134	1.0
Car/Truck	0.142	1.4	0.082	1.8
Phone (landline or cell)	0.204	1.0	0.120	1.2
Watch/Clock	0.212	1.0	0.149	1.4
Solar panel (typically to charge phone)	0.237	1.2	0.133	1.0
Animal drawn cart	0.342	1.2	0.242	1.1
Wheelbarrow	0.320	1.0	0.224	1.0
Cattle	0.349	1.9	0.243	1.2
Goats or sheep	0.254	1.9	0.165	1.6
Chicken or other poultry	0.233	1.6	0.154	1.5
*Additional assets*				
Stove stand			0.132	1.8
Bed			0.208	1.3
Mattress			0.107	1.6
Table			0.230	1.3
Chair			0.226	1.3
Trunk/suitcase			0.096	1.9
Lamp			0.089	1.0
Iron			0.208	1.0
CD player			0.154	2.0
Sewing machine			0.150	1.1
Plough			0.252	1.4
Cultivator			0.200	1.1
Hoe or spade			0.091	2.0
Pick			0.158	1.5
Mortar and pestle			0.209	1.7
Reed mat			0.126	1.9
Reed basket			0.176	1.5
Smoothing stone			0.176	2.0
Clay pot			0.097	1.9
*Water and sanitation*				
Water source: Piped			0.042	1.4
Water source: Protected			0.059	1.4
Water source: Unprotected			-0.077	1.8
Flush toilet or VIP/Blair latrine			0.129	1.5
Pit latrine			0.081	1.0
Mean item complexity		1.4		1.5
Proportion of variance explained by first principal component, %	20.7		16.6	

The averages for each housing characteristic and asset included in the index increased monotonically across quintiles from the lower to the upper. Linear trend test p-values were all <0.001. (Table 5). Characteristics, assets and all other economic measures not included in the construction of the index that represent better conditions also exhibited a pattern of increasing means from lower to upper quintile. Linear trend test p-values were, similarly, all <0.001. (Tables 6 and 7). Indicators that represent poorer conditions, such as unprotected water source and coping strategy indicators, had decreasing means from lower to upper quintile.

**Table 5:** Percentage of households possessing each asset included in the SHINE index across quintiles of the SHINE wealth index^1^.

	Wealth Quintile					
Item	Lower	Lower middle	Middle	Upper middle	Upper	N
Higher quality floor material	14.0	34.2	46.0	68.1	88.8	4,590
Higher quality wall material	42.5	59.7	66.3	73.8	87.5	4,595
Higher quality roof material	2.6	7.4	8.9	15.0	28.7	4,599
Electricity	1.7	4.2	7.5	10.0	13.7	4,649
Radio	29.2	57.3	74.4	86.4	95.2	4,657
Television	4.2	12.8	28.9	44.1	74.4	4,656
Bicycle	8.9	26.6	38.1	52.9	66.6	4,657
Car/Truck	0.3	1.3	1.3	3.2	15.0	4,646
Phone (landline or cell)	67.7	88.7	94.3	97.3	99.5	4,662
Watch/Clock	1.2	4.2	9.4	14.6	38.1	4,654
Solar panel (typically to charge phone)	28.2	57.6	74.4	80.2	86.2	4,655
Animal drawn cart	1.0	7.3	21.3	44.8	86.2	4,658
Wheelbarrow	11.0	25.8	47.2	69.2	90.6	4,657
Cattle	9.5	35.5	61.6	83.4	97.2	4,658
Goats or sheep	16.8	38.7	57.1	69.3	79.8	4,658
Chicken or other poultry	48.0	74.3	85.7	90.6	96.6	4,657

^1^ Linear trend test p-values were all <0.001

**Table 6:** Percentage of households possessing each asset NOT included in the SHINE index across quintiles of the SHINE wealth index^1^.

	Wealth Quintile					
Item	Lower	Lower middle	Middle	Upper middle	Upper	N
Stove stand	62.3	82.6	91.3	93.9	97.1	4,664
Bed	34.0	67.4	83.4	95.4	99.1	4,660
Mattress	12.9	23.3	29.9	38.0	48.3	4,657
Table	5.3	20.1	37.0	59.8	88.4	4,659
Chair	4.3	19.6	35.8	55.0	86.0	4,658
Trunk/suitcase	67.3	79.1	84.9	86.0	94.7	4,657
Lamp	13.3	17.5	23.9	27.4	40.9	4,655
Iron	14.4	31.4	57.5	71.0	88.5	4,655
CD player	8.6	19.9	28.4	39.5	60.4	4,657
Sewing machine	1.2	2.0	6.0	12.1	35.1	4,655
Plough	6.7	27.7	54.1	79.1	95.7	4,655
Cultivator	0.4	2.3	9.4	24.3	55.3	4,645
Hoe or spade	88.8	98.5	98.8	99.9	99.9	4,655
Pick	31.3	54.8	66.2	75.5	88.2	4,639
Mortar and pestle	8.5	22.5	41.4	62.4	81.3	4,659
Reed mat	48.1	61.7	69.9	78.4	90.0	4,658
Reed basket	19.1	40.5	49.9	65.2	84.5	4,655
Smoothing stone	17.9	33.7	51.4	65.7	80.6	4,651
Clay pot	62.2	77.5	82.0	84.9	91.3	4,646
Water source: Piped	33.0	39.6	42.6	45.3	46.8	4,958
Water source: Protected	14.4	19.0	19.6	22.3	32.9	4,958
Water source: Unprotected	39.9	32.5	29.1	24.3	14.9	4,958
Flush toilet or VIP/Blair latrine	10.1	15.4	17.7	29.9	49.0	4,951
Pit latrine	5.5	9.9	15.4	20.0	26.3	4,951

^1^ Linear trend test p-values were all <0.001

**Table 7:** Distribution of assets NOT included in the SHINE index or Expanded SHINE index across quintiles of the SHINE wealth index^1^.

	Wealth Quintile					
Item	Lower	Lower middle	Middle	Upper middle	Upper	N
Any formal salary/wages in HH, %	11.2	11.5	14.7	17.6	22.1	4,656
Income last month–Total, $U.S, Mean (SD)	77.1 (167.7)	114.1 (244.6)	142.4 (264.0)	184.2 (385.9)	288.6 (458.7)	4,656
Expenditures last month, $U.S., Mean (SD)	94.9 (166.5)	127.3 (163.4)	155.7 (204.7)	202.7 (306.5)	273.3 (341.2)	4,657
Coping strategy index, Mean (SD)	10.8 (16.6)	8.6 (15.0)	5.7 (11.5)	3.9 (7.6)	2.7 (8.0)	4,541
Any coping strategy, index >0, %	66.3	59.6	52.4	45.9	35.8	4,541
HH dietary diversity index, Mean (SD)	3.8(1.5)	4.1(1.5)	4.1(1.6)	4.4(1.6)	4.7(1.6)	4,548
HH meets minimum dietary diversity > 4 (%)	28.9	36.2	36.2	44.8	51.2	4,548

^1^ Linear trend test p-values were all <0.001

In our first sensitivity analysis, using PCA with an expanded set of 40 variables, the scree plot shows the first principal component as dominant (Fig 1B), explaining 17% of the variation. The overall mean item complexity was 1.5 supporting adequacy of model. After fitting a model retaining two principal components, the predicted wealth index scores based on the first principal component suggest an approximately symmetric, and normal, distribution (Fig 1D). Loadings for this component all had the expected sign although more than one half had absolute loadings less than 0.2 (Table 4). The median loading was 0.15 (IQR, 0.10–0.19).

There was strong evidence of a positive correlation between the core and the expanded SHINE wealth indices. The Spearman rank correlation coefficient was 0.910 (95% CI: 0.903–0.921). There was 60% agreement between the indices grouped into quintiles and the linear weighted kappa statistic for the predicted quintiles was 0.725 (95% CI: 0.713–0.736), indicating substantial agreement [38] (Table 8). Agreement was higher when comparing indices grouped into quartiles or terciles.

**Table 8:** Sensitivity analysis and agreement with SHINE wealth index.

Index Comparison	% agreement with SHINE wealth index	Weighted Kappa (95% confidence interval)
Expanded SHINE wealth index		
Tercile	76.2	0.730 (0.714–0.743)
Quartile	68.0	0.734 (0.723–0.747)
Quintile	59.7	0.725 (0.713–0.736)
Polychoric PCA index (using core variables)		
Tercile	96.1	0.956 (0.950–0.963)
Quartile	95.4	0.963 (0.958–0.968)
Quintile	93.8	0.961 (0.957–0.966)

The second sensitivity analysis used polychoric PCA on the set of 16 core variables. The first principal component accounted for 32% of the variation and all loadings were positive. The scree plots and histogram of the derived wealth index showed patterns similar to Fig 1A and Fig 1C. The Spearman rank correlation coefficient was 0.910 (95% CI: 0.904–0.915) and there was 94% agreement between the quintiles and the linear weighted kappa statistic was 0.961 (95% CI: 0.957–0.966), indicating almost perfect agreement. Agreement was even higher when comparing indices grouped into quartiles or terciles. Using the expanded variable set for polychoric PCA yielded similar results (not shown).

**Fig 2:**
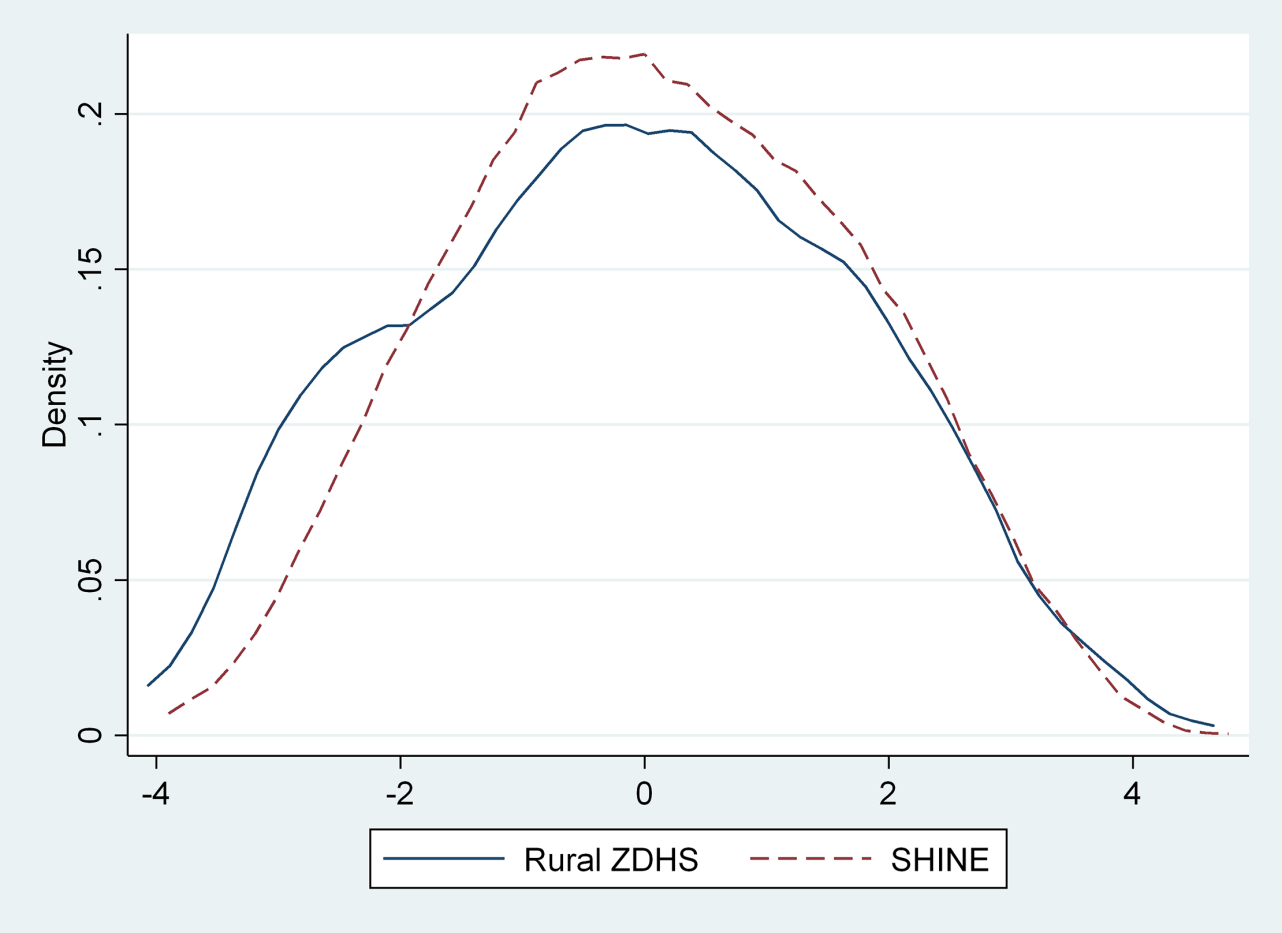
Comparison of distribution of index scores between rural households included in the 2015 Zimbabwe Demographic and Health Survey (ZDHS) and SHINE households.

In our final analysis based on a PCA using the selected 15 binary variables and all rural households from the 2015 ZDHS, we found good correspondence with the DHS-constructed index provided with the data (with Spearman rank correlation coefficient of 0.862 95% CI: 0.854–0.869] and a linear weighted kappa statistic for indices grouped as quintiles of 0.663 [95% CI: 0.652–0.674]). The distributions of index scores for the two samples generated with this common set of weights have nearly perfect common support (Fig 2). Households in SHINE were modestly wealthier than the overall population of households in rural Zimbabwe though the average index score was only 0.1 SD higher in SHINE and not significantly different (p = 0.10). What difference there is derives from a slight excess of less wealthy households in the full ZDHS compared to those in SHINE, while the distributions are nearly identical in the higher, wealthier tail. Results were similar when we redid the analysis using only rural households from Midlands Province, the lowest level at which the DHS is representative.

## Discussion

Using 16 items, the SHINE wealth index based on the first principal component performed well—it explained 21% of the total variation, had all positive loadings on the items, and did not exhibit substantive truncation or clumping. Examining across quintiles of the index (from lower to upper), average values of each component item increased significantly and monotonically in quality, as did a number of other assets and economic measures not included in the index, providing evidence of both internal and external validity of the index. These included measures of income and expenditures over the last month, inappropriate for direct inclusion in the index given the relatively short recall period and different timing of the baseline surveys, but nevertheless providing additional evidence that higher index scores were positively associated with greater economic resources.

A comparison of the extent to which the index categorized relative wealth of members of the study population similarly to categorizations based on measures constructed using alternative approaches indicated substantial or almost perfect agreement. These included PCA using an expanded set of household characteristics and polychoric PCA using the core set of variables. Agreement between alternative approaches was slightly weaker for the modification to the variable set in contrast to the modification in the estimation approach, as also reported by Howe et al (2008)[17]. From these two sensitivity assessments, we concluded that the SHINE wealth index is adequately robust, supporting our strategy of using a more limited core set of variables.

We defined all variables in analyses to be binary and therefore did not consider MCA. Without a strong rationale for assuming a latent causal model underlying wealth, we also did not consider FA.

A related wealth index constructed using 2015 ZDHS rural households, and applied to SHINE households, demonstrated that the SHINE sample has a similar, though modestly higher average wealth index than other households in rural Zimbabwe.

The study had some limitations. First, there was no “gold-standard” measure of full expenditures or income against which to validate the indices. Second, 11% (576 of 5,280 enrolled) of baseline surveys were never completed and all analyses necessarily exclude those households. Observed differences in some demographic characteristics between those who completed a baseline survey and those who did not may have led to some selection bias. Third, while agreement among categorizations was good when comparing alternative approaches, it was not perfect, leaving the possibility of misclassification errors in analyses using quantiles.

## Conclusions

Measuring wealth in a randomized, controlled trial like SHINE is important for a number of reasons, including quantifying inequities, making statistical adjustments for confounding variables and examining effect modification. However, there is no universally agreed-upon approach to such measurement. In this paper, we developed and validated a household wealth index using baseline data for the Sanitation, Hygiene, Infant Nutrition Efficacy Trial conducted in rural Zimbabwe between 2012 and 2017 [11]. In community-randomized trials with a small number of clusters, creating an index has the added benefit that the analyst does not lose as many degrees of freedom as the alternative approach of controlling for multiple factors.

Building on the literature and considering the variables important in the local context and to study design (for example excluding variables directly targeted by the intervention), we compared the index to potential alternatives. We find that a “standard” approach (principal components analysis) using a rich, but still relatively parsimonious set of variables is strongly associated with a wide range of indicators of wealth—and is both internally and externally valid. Moreover, an expanded variable set or alternative estimation approach only minimally changes the variation described by the index. From these assessments, we conclude that the SHINE wealth index is adequately robust. We then conducted PCA on all rural households in the 2015 ZDHS to enable a comparison of the distribution of wealth in the two samples using a single common set of weights. In addition to providing evidence of the validity of the index, the paper provides a template for others constructing such indices, including a method for placing smaller regional samples into the broader context of a country when national survey data are available.

The results, however, do not imply that the SHINE wealth index is without measurement error. For example, there are possible misclassification errors in the quantile classifications of wealth made using the index, even though the proportion of explained variance exceeds 20% [45]. In analyses where the role of wealth is likely to be highly relevant, analysts may want to consider variations of the index (e.g., employing directly the index value instead of derived quantiles or considering different quantiles since agreement was higher for terciles compared to quintiles) or, on occasion, include directly some of the important underlying characteristics.
